# Patients understanding of depression associated with chronic physical illness: a qualitative study

**DOI:** 10.1186/1471-2296-15-37

**Published:** 2014-02-20

**Authors:** Sarah L Alderson, Robbie Foy, Liz Glidewell, Allan O House

**Affiliations:** 1 Institute of Health Sciences, University of Leeds, Leeds, UK

## Abstract

**Background:**

Detection of depression can be difficult in primary care, particularly when associated with chronic illness. Patient beliefs may affect detection and subsequent engagement with management. We explored patient beliefs about the nature of depression associated with physical illness.

**Methods:**

A qualitative interview study of patients registered with general practices in Leeds, UK. We invited patients with coronary heart disease or diabetes from primary care to participate in semi-structured interviews exploring their beliefs and experiences. We analysed transcripts using a thematic approach, extended to consider narratives as important contextual elements.

**Results:**

We interviewed 26 patients, including 17 with personal experience of depression. We developed six themes: recognising a problem, complex causality, the role of the primary care, responsibility, resilience, and the role of their life story. Participants did not consistently talk about depression as an illness-like disorder. They described a change in their sense of self against the background of their life stories. Participants were unsure about seeking help from general practitioners (GPs) and felt a personal responsibility to overcome depression themselves. Chronic illness, as opposed to other life pressures, was seen as a justifiable cause of depression.

**Conclusions:**

People with chronic illness do not necessarily regard depression as an easily defined illness, especially outside of the context of their life stories. Efforts to engage patients with chronic illness in the detection and management of depression may need further tailoring to accommodate beliefs about how people view themselves, responsibility and negative views of treatment.

## Background

Co-morbid depression and chronic physical illness is associated with increased morbidity and mortality [1,2] although detection and management rates in primary care are well below stated prevalence’s [3]. Our limited knowledge of how people with chronic illness understand depression is likely to contribute to this gap as it hinders our ability to tailor detection and clinical care strategies to their needs [4]. Patient beliefs are known to influence engagement with the detection of other illnesses such as cancer [5] and diabetes [6]. Furthermore, it is recognised that effective management of a variety of conditions in primary care depends upon understanding and responding to patients’ ideas, concerns and expectations [7,8]. For example, patients with back pain are often reluctant to undertake therapeutic physical activity because of fears about exacerbating underlying structural problems and pain related fears are related to engagement with management and long term disability.

Screening has been proposed as one intervention to increase detection. In the UK it is recommended by national guidance and was, until recently, operationalized via a financial incentive scheme and the inclusion of depression associated with chronic illness in the Improving Access to Psychological Therapies programme [3]. The Canadian Task Force on Preventative Care and the U. S. Preventive Services Task Force also encourage screening for depression, but only where there is collaborative care available to ensure structured follow up and management [9-12].

We explored patient beliefs about the nature of depression associated with physical illness, especially the degree to which those beliefs conform to ideas about depression as an illness.

## Methods

We developed a topic guide that included open-ended prompts to elicit personal experiences of depression and depression related to chronic physical illness and then more structured questions to explore themes identified in an earlier systematic review [4] of patients understanding of depression that drew upon the Common Sense Self-Regulatory Model Illness Representations as a guiding framework [13].

Semi-structured interview schedule

•Some patients with diabetes/CHD* become depressed. Why do you think this is? Have you any experience of this? (*diagnosis of participant)

•What do you think depression is?

•What causes depression?

•How can depression be cured or controlled?

•How long does depression last?

•Does the depression have any consequences for you/others around you?

•Does depression change you?

•Does having depression change the way others see you?

•How well do you understand depression?

We invited participants from two urban general practices in Leeds, UK, with differing deprivation indices (IMD 24.8 and 39.4, Leeds PCT mean 25.8). Patients with coronary heart disease and/or diabetes were systematically sampled from each practice. These disease registers were chosen due to depression screening being incentivised in these diseases within the UK at the time of the research. A second round of recruitment took place to obtain participants of ethnic groups other than White British by asking GPs to distribute recruitment packs opportunistically during face-to-face consultations. We aimed to continue recruitment until no new avenues of enquiry were identified.

Interviews took place at venues preferred by participants. Interviews were audio recorded and observational notes taken. Participants completed the Hospital Anxiety and Depression Scale (HADS) [14] at the start of the interview to ascertain current depressive symptoms and were asked to disclose any known current or past personal experience of low mood. We chose the HADS because it gives a more conservative estimate of the presence of depression in patients with co-morbid disease [15].

Data were transcribed verbatim, anonymized and verified for accuracy, then uploaded in to NVivo8 for analysis. Thematic analysis involved a constant comparison process, aligning new data with existing codes, generating new codes, reviewing earlier transcripts for new codes, grouping data into major and subthemes, and identifying negative cases. As analysis proceeded, and emerging findings were discussed, we perceived a risk that our thematic analysis excluded important contextual information and the relevance of the life story; hence the analysis was expanded by looking within each transcript for continuity and relevant contextual information.

The research team discussed the coding process and the development of themes throughout the analysis process. The interviewer (SA, a GP) kept a reflective journal to aid analysis. The researcher’s role as a GP and its influence on the interviews was reviewed with the aid of the reflective journal.

## Results

Twenty-six people took part in interviews for this study, mostly at the participant’s home or GP surgery. Participants were mostly white British males not in work (Table 1). Seventeen participants reported past or current low mood, and eight participants had HADS scores compatible with mild to moderate depression at the interview, with only those scoring moderate depression self-reporting current low mood and those scoring mild depression reporting past depression only.

**Table 1:** Demographics of participants interviewed about beliefs about depression associated with chronic physical disease

		**Participants**
**Gender**	Male	22 (84.6%)
**Age**	Mean Age	65.1 (range 45–89)
**Ethnicity**	White British	23 (88.5%)
	White Other	1 (3.8%)
	Asian Indian	1 (3.8%)
	Asian Pakistani	1 (3.8%)
**Working**	Employed	6 (23.0%)
**Chronic disease**	Diabetes	9 (34.6%)
	CHD	12 (46.2%)
	Diabetes & CHD	5 (19.2%)
**History of low mood**	Yes (at the moment)	2 (7.7%)
	Yes (in the past)	15 (57.7%)
	No, never.	9 (34.6%)
**HAD score**	None	18 (69.2%)
	Mild	6 (23%)
	Moderate	2 (7.7%)
	Severe	0 (0%)

**Figure 1:**
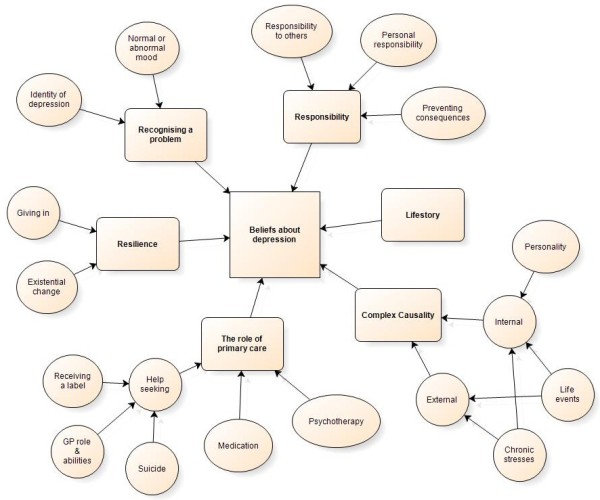
Themes and sub-themes of patients’ beliefs about depression associated with chronic physical illness.

Six main themes were developed that reflected how participants described depression. The themes are presented in two categories of beliefs; those that fit with conceptualising depression as an illness, and those that do not; the theme of life story is compatible with both. Figure 1 illustrates the 6 main themes and the sub-themes.

### Recognising a problem

Participants described depression as a change into an abnormal state and different to a ‘normal’ low mood due to its debilitating nature. Level of symptoms alone was relevant, so that only those who scored moderate depressive symptoms on the HADS regarded themselves as having an abnormal state, whereas mild depression on the HADS was seen as not severe enough to be abnormal.

Those with experience of depression had not recognized that they had developed an abnormal state and struggled to view it as an illness. People around them noticed sooner and had to encourage them to seek help.

It’s an horrendous illness, it’s shocking, you feel totally useless, you feel that you’re no use to your family, you’re no use to your children, you’re no use to yourself, you can’t drag yourself out of it, you can’t be happy, you can’t do anything. Participant 20, male CHD, past depression

Don’t get me wrong I’m still taking depression tablets, but I’m not depressed, well touch wood, I don’t think I am anyway. Participant 15, male diabetes, past depression

Well I never noticed it. Everybody else noticed that my mood had changed, I were more, not moody, but dark. If sommat wasn’t right I used to really lay into whoever it was. Participant 22, male CHD & DM, past depression

### Complex causality

All participants recognised chronic illness as a cause, with diagnosis often being a life-changing event that forced people to face mortality and potential disability and were often seen as interlinked. Physical illness was a persistent source of stress to some, with difficulties in understanding how to manage it and feelings of guilt when not following lifestyle restrictions. Suggested explanations about why some individuals were more vulnerable than others included the timing of the diagnosis of depression related to that of the chronic disease, the familiarity of the chronic disease being diagnosed (for some, family members with the same chronic disease reduced worry about the future) and the ongoing impact the chronic disease had upon the participant’s life.

Other life events (such as bereavement or relationship breakdown) were justifiable causes of depression; however chronic stresses (such as unemployment and family problems) were not justifiable as they could be resolved by action from the sufferer. A few participants mentioned a biological cause to depression, but none used it to explain their own depression, preferentially explained by external events.

I can understand people with heart disease or whatever do get fed up because ultimately, if you think about it in its blackest terms, it is probably going to kill you in the end, you are going to die from it or a subsidiary of it or it’s going to disable you to some degree and I can understand some people sat there thinking “well what now?” Participant 4, male CHD, past depression

I’m worried about this diabetes and I mean there’s nothing I can do about it apart from keep taking the tablets and that eliminates 90% of the worry if you like, but it’ll always be there, worry, it’s constantly on my mind I’ve got to think right next week I’ve got to make a doctor’s appointment for 2 weeks ahead, you know what I mean. Participant 11, male DM, no depression

It got through that chink in the armour, which we all have, you know, there’s always something that gets to you, I honestly thought to be honest that I was stronger than what I thought, you know and when she said you’re suffering from depression, I thought I’m not, no way! Participant 22, male CHD and diabetes, past depression

All he needed was to change his lifestyle and change his situation, to stop feeling sorry for himself and just to move on and you know. Participant 10, male diabetes, no depression

### The role of primary care

Participants’ opinions varied about whether help should be sought from a doctor. Those without depression felt it was not the doctors’ role and that GPs were unable to help with situational causes, such as increasing social contact and support. Only when suicide was being considered should help from the GP be sought. Those with depression felt primary care was appropriate for seeking help and described positive experiences with help being offered which had enabled their recovery.

There were conflicting views as to whether the label ‘depression’ should be used. Participants both with and without depression felt it could be harmful and stigmatising. Others considered it helpful and reassuring to have a diagnosis; knowing what was wrong could support recovery.

The participants taking medication for depression saw it as a control method rather than a cure, whereas participants with no history of depression viewed medication negatively and saw it as a mask rather than treating the cause. Psychotherapy was viewed positively by those who hadn’t experienced depression personally, it would help solve the problem and be preferable to medication, but negatively in those who had tried it. They found the treatment differed from what they expected and did not resolve the cause (social problems).

I wanted to get back to work and I wanted to get normal and I don’t think, without the medication to keep my mood down, not down in a bad way, but to keep me on a level plane and compos mentis, if you want for a better way of describing it, I think it’d have taken a hell of a lot longer, without that because it keeps you on a level par, but I think if I hadn’t taken it I wouldn’t be where I am now back at work. Participant 22, male diabetes & CHD, past depression

I think the things that could really help are not available to doctors, you can’t make somebody go and have a coffee morning with some friends, you can try and do them self-help groups and stuff like that but you can lead a horse to water but you can’t make it drink can you, I think they’re the real things that help people turnaround. Participant 10, male DM, no depression

I spoke to the GP, they wanted to put me on pills which I didn’t want to take pills so got me on this CBT, cognitive behavioural therapy and the chap that I was dealing with, although he was a nice enough chap and stuff, I don’t think in any conversation I had with him there was anything searching or questioning from him which I was really cynical about, I expected some kind of, I mean as we are doing, kind of talking about my life post heart attack, you know, which includes times when I’ve been down and there was nothing like that from him which after a while, being cynical anyway, I just thought it was a waste of time. Participant 25, male CHD, past depression

### Responsibility for depression

Those with experience of depression felt an individual responsibility not to allow the consequences of depression to affect the family and home, and under pressure to recover in order to return to work as the family wage earner which encouraged help-seeking.

Many participants believed in a personal responsibility for resisting depression or to make the changes needed to get better. Not taking responsibility to look after oneself was seen as a personality flaw and for some, a reason to look down upon those who have depression. There was an internal responsibility to take control of the situation and make the changes needed to work towards a cure. This was in contrast to help-seeking from others which was viewed as being an easy mask for the real problem. Suicide was seen as taking responsibility for some, with the act being a method of ending the suffering of others around the person.

[My wife] got angry at times and she got angry with me because she could see that I wasn’t my usual self and the normal me wouldn’t be like that and that’s what angered her, she wanted me back. Does that make sense? Did she didn’t want this one, she didn’t like this one and this is, you were asking me earlier about what I do and what I think, I tend to keep my thoughts way in now because I don’t want her to see that not very nice me . Participant 4, male CHD, past depression

No, you know, I think it’s you have to change yourself instead of taking responsibility of yourself you should change yourself, that it should not happen again. Participant 12, male CHD & DM, no depression

### Resilience

Several participants believed they became depressed only when they ‘gave in’ to their low mood. The causal factors and symptoms were there, but the individual had to succumb to them in order to be depressed. Resilience was described as an entirely individual characteristic rather than including a social network from which to draw strength. Whilst family and friends were often mentioned in the recovery from depression, initial vulnerability depended upon individual’s own resources. Resilience was personality driven with both weak and strong people becoming depressed if they had cause, however weaker personalities would succumb faster.

Some participants struggled with the depression diagnosis as it caused them to re-evaluate how they saw themselves, having never felt they were the ‘type’ of person to get depression. They no longer recognised themselves and it changed their sense of social and individual identity, causing distress and self-dislike.

Oh yeah, anyone can get it, yes, yeah but I’ve still got to be careful not to look down on them, in fact my friend who I mentioned, he is a manic depressive, his mother is also a similar…. and I always thought myself a bit superior, I know I shouldn’t, I know I shouldn’t because it’s wrong but I feel glad, not glad that I’m not like that but a bit upperty because I’m not like that. Participant 18, female DM, no depression

My dad suffered depression but because my mum was in a worse state he didn’t give in to it. He still suffered from it but if he had given in there would have been no one to look after the kids. Participant 1, male DM, current depression

I’ve always been a strong woman, so strong, I’ve had to be, not having any parents from like 16 so I had to be and now I feel like I’m not. Participant 29, female CHD & DM, current depression

### Understanding the life-story

The idea that individuals could not explain depression without telling their life story was a common theme throughout all the interviews and fits with beliefs both compatible and incompatible with depression as an illness. Participants were unable to discuss the illness as a discrete part of their life. They used their life story as an essential part of their explanation of why they had or had not become depressed. Some participants who struggled with the concept of depression answered all questions by detailing parts of their life without talking about depression at all. The life story was not seen as causal, but as a way of understanding and explaining themselves as people.

Participant 6 vignette:

Topics discussed included his previous employment, his family, holidays, his volunteer work and beliefs about his coronary heart disease. He was unsure whether a GP should be consulted for mood problems and quickly changed the subject to a leg injury he suffered. He appeared to be uncomfortable with any questions related to mood and described his life in detail in response to questions about depression. Participant 6, male CHD, no depression.

## Discussion

Six types of belief appear important to explore in the detection and subsequent management of depression in people with chronic illness; recognizing a problem, complex causality, the role of primary care, responsibility, resilience, and the importance of the patients’ life story in feeling understood.

•Patients do not necessarily understand their undiagnosed distress as 'depression' which makes recognition difficult and explains why they may not present to primary care.

•Patients identify a strong link between chronic physical illness and depression that was common throughout all the interviews although there were differences in perceived strength of the link. Discussing depression in a chronic disease review may lead patients who perceive a weak link between the two to not disclose in this situation. Patients’ complex reasons for feeling low may need to be identified so that social factors can be explored, rather than just concentrating on the link between ill health and depression.

•Fears about stigma and taking medication for a situational problem may also stop patients disclosing distress.

•The sense of responsibility for depression, either in not succumbing to the causes or changing the circumstances to resolve the problem, is an important finding that requires personal action rather than seeking medical treatment.

•Depression is perceived to alter a person’s self-identity and not wanting to accept this change may lead to patients avoiding an identity-changing diagnosis. Resilience to depression is seen as an individual trait rather than being derived from social networks.

•Patients value time to tell their life story in order to justify why they have depression.

### Strengths and limitations

We believe this is the first study to specifically explore beliefs and experiences of depression in a population with chronic illness targeted by depression screening.

The first author’s role as a GP was disclosed at the interviews and may have influenced participants’ interactions. The reflective journal suggested that participants used the interview as an opportunity to get a medical professional to understand their point of view, something that was difficult in a time-limited consultation.

The interview schedule was informed by our preceding systematic review; [4] however, by asking the participant to describe their personal experience of depression using open-ended questions we ensured participants had opportunity to describe their beliefs in detail before prompts were used.

Participants were not sampled based upon diagnosis of depression as this study aimed to explore beliefs about depression in those targeted by an initiative to detect depression. Both patients with and without experience of depression contributed to the majority of themes, although their views differed greatly concerning the role of primary care.

Despite efforts to recruit a diverse sample, particularly more females and ethnic minorities, the participants were mostly white British males. This is likely to be due to the higher prevalence of CHD and diabetes in males. It is possible that patients of different ethnicity might hold different views, however, previous studies suggest that this may be a difference in emphasis rather than absolute differences in views [4].

We selected diabetes and coronary heart disease as chronic diseases as they are targeted by initiatives to detect depression. However, many of the issues we identified are likely to be relevant to other chronic diseases such as chronic obstructive pulmonary disease or arthritis.

With this qualitative approach, patients’ beliefs about depression were explored. Conclusions cannot be drawn regarding actual behaviour and engagement with detection; however, assessment of behaviour fell outside the scope of this study.

### Comparison with existing literature

This study provides new insights into why patients with CHD or diabetes may not present or engage with detection for depression within primary care. Several studies relating to beliefs about depression associated with chronic physical illness exist; [16,17]; however, none has explored depression in this targeted population or beliefs about depression beyond those only associated with chronic physical disease [4,16,18]. One of the important findings in this study is that beliefs about depression in general cannot be separated from beliefs about depression related to chronic physical illness, the two are interlinked in an individual and both need to be taken into account. Previous studies on illness beliefs in patients with multi-morbidities also found that beliefs about individual illnesses were affected by multi-morbidity [17].

This study found that patients share similar beliefs to clinicians, viewing depression as an understandable reaction to chronic disease and a social cause to depression. Patients also found it difficult to recognise the symptoms in themselves, often normalising the problem [18].

The idea of a disease changing the self-identity of a person has been researched in other illnesses [19-24]. These studies identified a loss of self and a shift in identity to a person with a disease or a survivor or victim which can affect coping and the impact of diagnosis. Our findings suggest that depression is also associated with a change in individuals’ perceptions of themselves and their roles. We cannot be sure that this strong sense of altered self is attributable to depression or is displaced from the sense of altered self that comes from chronic disease.

Kangas [25] and Kokanovic [26] have found that patients use a life story to talk about their depression, often absorbing it into their story to reinforce that they had always been depressed. In this study patients used the life story to explain why they did not have depression and how their life experience helped them recover, rather than seeing potential recovery as a threat [26]. Our participants described their depression in the context of their life story, apparently mainly as a way of contextualising their current experiences rather than because they always had a life narrative into which depression was woven. Needing time to tell a life story will be difficult to resolve in primary care where consultations are time limited, although continuity of care with one practitioner may help and is often seen as important by those with chronic illness and the elderly for this reason [27]. The emphasis of services for mental health is currently shifting from a focus on explanation in terms of the self-identity of the patient towards coping skills development (e.g. brief interventions offered by Improving Access to Psychological Therapies). Yet our and others’ work suggest patients prefer an opportunity to discuss their lives more widely. This may relate to the finding that those who had experienced brief psychological interventions did not feel it addressed their needs whereas it was viewed positively by those with no personal experience. There may be limited understanding of what psychotherapy entails leading to unmet expectations. Psychological interventions have small effects only on depression symptoms in patients with coronary heart disease and chronic obstructive pulmonary disease [28,29]. Unmet patient expectations may help explain this finding.

The difficulty individuals have in recognising their distress or a change to an abnormal state has been identified in other studies [18,30], and the reluctance to visit their GP with what might be considered a minor problem compared to a physical one such as heart disease, or with failing to cope with life, has also been identified previously [18,31]. Negative beliefs regarding medication and stigmatization have been identified previously as barriers to recognising depression associated with chronic physical disease [18]. We also found an additional barrier in that depression negatively changed the sense of social and individual identity of the patient and prevented admission of depression symptoms.

We found other factors that can facilitate disclosure of depressive symptoms. Those with depression believed that despite the chronicity of their own depression, the positive view of primary care and the help received there by those with depression offered hope for recovery. Depression has a mean duration of 18 months in those over the age of 55 in a recent study [32] and this hope for recovery may facilitate help-seeking from primary care in this age group.

Literature describes resilience in managing or resisting depression as drawing on personal resources (‘being strong’) and deriving support from the network of people around the depression sufferer [33]. The participants in our study largely interpreted resilience as an individual attribute. This may explain why patients in the study by Dowrick et al. study preferred to manage their mental health with personal approaches such as drawing on personal strength rather than support from others [34].

Viewing help-seeking as not taking responsibility for addressing the circumstances that cause the depression may prevent disclosure of depression symptoms. Identifying such beliefs in consultations may be important in depression recognition and management in consultations about chronic disease.

### Implications for practice and research

Further work is needed to develop tailored detection strategies that better fit with how patients think. When approaching a new diagnosis, the acceptance by the patient often involves a process of assimilation of information and accommodation to existing beliefs. Experience with other conditions, such as dementia and cancer, suggest that the sharing of potentially difficult diagnoses is seldom a ‘one-off’ process [35,36]. The detection of depression associated with chronic physical illness in primary care in the face of multiple competing demands is therefore a more complex task than previously appreciated [37]. The findings from this study suggest that doctors will need to be flexible in consultations and explore patients’ ideas, concerns and expectations when detecting depression. Questions aimed at uncovering these beliefs may help patients to disclose their concerns. The following list provides examples of issues that could be explored in consultations to elicit these beliefs before approaching screening.

•The difficulty and uncertainty in managing a chronic condition on top of other life pressures – is this manageable or the last straw?

•The difficulty people have in recognising depression in themselves although others may have noticed a change in mood

•Concerns about not being able to function as normal – what can people not do anymore that they used to be able to do?

•People with depression often do not feel like themselves anymore

•Concerns about being a ‘weak’ person by admitting to depression symptoms

•Concerns about the effects of treatment and its ‘masking’ of the problem

•Feeling responsible for being depressed and not being able to change the situation

Given the accumulating evidence [1,2] showing that depression associated with chronic physical illness has a detrimental effect on morbidity and mortality, health professionals will continue to be encouraged to actively seek such at risk people. The list summarizes summarises implications of our findings for mental health care provision.

•Care needs to be taken when asking about possible depression in chronic disease reviews so that those who are symptomatic but perceive a weak link between the two feel able to disclose.

•The responsibility felt by those with depression for not being able to resist depression could be reduced by explaining the link between depression and chronic disease before case-finding.

•Concerns regarding medication ‘masking’ the problem and adding to the tablet burden might be improved by increasing access to services such as brief psychological interventions (such as IAPT) at the time of referral.

•Integrating chronic disease and mental health services (such as in collaborative care models) may help reduce the stigma of depression and the burden of extra appointments.

•Unmet expectations following psychotherapy may be prevented by discussing the role of brief interventions (such as IAPT) to increase understanding.

## Conclusion

People with chronic physical illness hold a range of beliefs about depression which may help explain some under-detection in primary care. An understanding of patients’ reasons for presenting or not disclosing distress may assist in identifying subgroups of patients with different management needs, facilitate the targeting of GPs’ time and therapeutic efforts, and guide more individualised care.

### Ethical approval

Ethical approval was obtained from South Yorkshire NHS Research Ethics Committee (REC reference 10/H1310/81).

## Competing interests

The authors declare that they have no competing interests.

## Authors’contributions

SA was responsible for the study conception and design, data collection and data analysis. RF, LG and AH contributed to the data interpretation and drafts of the manuscript. SA will act as guarantor. All authors read and approved the final manuscript.

## Pre-publication history

The pre-publication history for this paper can be accessed here:

http://www.biomedcentral.com/1471-2296/15/37/prepub
